# Case report: manualized trauma-focused cognitive behavioral therapy with an unaccompanied refugee minor girl

**DOI:** 10.3402/ejpt.v7.29246

**Published:** 2016-01-14

**Authors:** Johanna Unterhitzenberger, Rita Rosner

**Affiliations:** Clinical and Biological Psychology, Catholic University Eichstätt-Ingolstadt, Eichstätt, Germany

**Keywords:** refugee, asylum-seeking, separated, adolescent, PTSD, trauma-focused, treatment, psychotherapy, manualized

## Abstract

**Background:**

There is uncertainty whether young traumatized refugees should be treated with culturally adapted psychotherapy or with an evidence-based western approach. As yet, empirical studies on culturally adapted treatments for unaccompanied young refugees in industrialized host countries are not available. Studies do, however, suggest that trauma-focused treatment is promising for this group.

**Objective:**

We describe the treatment of an unaccompanied refugee minor girl with posttraumatic stress disorder (PTSD) who underwent manualized trauma-focused cognitive behavioral therapy (TF-CBT; Cohen, Mannarino, & Deblinger, 2006).

**Methods:**

A 17-year-old girl from East Africa, who came to Germany without a caregiver, was treated for PTSD resulting from several traumatic experiences and losses in her home country and while fleeing. She lived in a group home for adolescents. Baseline, post, and follow-up data are reported.

**Results:**

The girl participated in 12 sessions of manualized TF-CBT. Her caregiver from the youth services received another 12 sessions in line with the treatment manual. Symptoms decreased in a clinically significant manner; at the end of the treatment, the girl was deemed to have recovered from PTSD. Treatment success remained stable over 6 months.

**Conclusions:**

Manualized TF-CBT is feasible for young refugees without significant cultural adaptations. It can, however, be seen as culturally sensitive.

Trauma-focused cognitive behavioral therapy (TF-CBT; Cohen, Mannarino, & Deblinger, 2006) is an evidence-based treatment manual for children and adolescents with posttraumatic stress disorder (PTSD). In line with treatment guidelines, a recent review (Leenarts, Diehle, Doroleijers, Jansma, & Lindauer, 2013; PTSD after childhood maltreatment) reported manualized TF-CBT to be the best-supported therapy for young people. While TF-CBT may be understood as a treatment approach *per se*, we are specifically referring to Cohen and colleagues’ manual in this paper. Recently, we reported on six unaccompanied refugee minors (URMs) who were treated with TF-CBT (Cohen et al., 2006) showing the feasibility of this approach for the group of URMs (Unterhitzenberger et al., 2015). Still, many clinicians are uncertain about manualized therapies for young refugees. We present this case report to illustrate how this treatment is applied to a URM patient in detail.

TF-CBT (Cohen et al., 2006) is a short-term psychotherapy involving twelve 45–50 min sessions with the child and the same number with a caregiver (usually a parent). The therapy comprises eight modules (acronym: PRACTICE): (1) psychoeducation with child and caregiver, teaching positive parenting skills, (2) relaxation, (3) affective modulation and emotion regulation, (4), cognitive processing, (5) trauma narrative, (6) *in vivo* exposure (if necessary), (7) conjoint child–caregiver session sharing the narrative, and (8) enhancing future safety (Cohen et al., 2006). TF-CBT is reported to be culturally sensitive in each module and was tested in culturally diverse settings beforehand (e.g., Murray et al., 2015). Murray, Cohen, Ellis and Mannarino (2008) name some components that make TF-CBT applicable in young refugees: its theoretical basis, skills-based approach, and short duration. Also, cultural modifications are possible without changing the treatment manual. Yet, only one study has tested its efficacy for refugee children (Schottelkorb, Doumas, & Garcia, 2012). However, discrepancies from the original manual were numerous (two to four parent sessions, no conjoint sessions), which hinders the evaluation of results.

Although the treatment recommendations for children and adolescents with PTSD are clear, young refugees are just starting to receive attention in traumatic stress research. Reviews of the literature on mental health interventions in this population (Eberle-Sejari, Nocon, & Rosner, 2015; Tyrer & Fazel, 2014) reveal major methodological limitations. Additionally, most treatment protocols have been studied only once. However, a trauma-focused approach seems promising (Eberle-Sejari et al., 2015). Although some authors argue convincingly that a culturally adapted application or a multimodal approach is especially necessary for refugees (e.g., Droždek, 2015), we were unable to find any evaluations of this for URMs in the literature. Given the range of cultural backgrounds in the refugee community in host countries, it seems difficult to decide *which* culture the intervention should be adapted to. This controversy leaves practitioners and researchers without any clear recommendations on how to treat young refugees suffering from PTSD. This is probably one of the reasons – in addition to language barriers and the problem of financing – why young refugees do not receive the psychosocial treatment they need, leaving this severely stressed group of URMs without sufficient access to mental health support (Bean, Eurelings-Bontekoe, Mooijart, & Spinhoven, 2006). However, not only because of mental health issues but also because of the possible negative influence of trauma symptoms on asylum decisions (Turner, 2015), the treatment of refugees with PTSD seems highly important.

## Objective

The aim of this report is to explain how manualized TF-CBT (Cohen et al., 2006) for a URM girl with PTSD worked in an outpatient setting. Furthermore, we give a detailed description of the therapy to examine differences in manualized TF-CBT between young refugees and non-refugees with a view to deriving treatment suggestions for the future.

## Case report

### Initial assessment

Amina was born in a country in East Africa. After her parents’ separation when she was an infant, her mother found it difficult to provide food and shelter for the family. At the age of 5, Amina was circumcised. She grew up in the country's capital where civil war has been raging for as long as she can remember. The family had to flee from their home to a camp several times. On one occasion, Amina saw a little girl being raped and killed in that camp. Three of Amina's siblings died violently. One brother was shot by a stranger after refusing to give him a cigarette, one was killed by gunfire on the street, and one sister died in a bomb attack. She saw the corpses. One day Amina's aunt decided to flee to Europe with her. In the Mediterranean, the boat capsized and Amina's aunt drowned. Amina was rescued and eventually reached Germany.

Seventeen-year-old Amina lived with eight other adolescents in a residential group of a youth welfare institution. Amina had been in Germany for 2 years and living in the group for 15 months. She attended a preparatory class (German and mathematics) for secondary school as she had had no schooling in her native country. Her German language skills were sufficient for psychotherapy.

Amina reported recurring nightmares, panic-like fright reactions as a consequence of intrusions, fear of loud noises, aggressive behavior, and self-mutilation. She could not look in the mirror as this triggered a panic reaction. The caregiver reported that Amina's thoughts were racing and she had no skills to regulate her emotions. Amina avoided going anywhere alone and had difficulties separating from her key caregiver in the residential group, a 56-year-old female social worker named Mrs. B. Furthermore, she had problems concentrating, difficulties in feeling emotions, and guilt regarding her family.

### Clinical measures

At intake, Amina was assessed using the Clinician Administered PTSD Scale for Children and Adolescents (CAPS-CA; Nader, Kriegler, Blake, & Pynoos, 1994). The cut-off for clinically significant symptomatology is 35. Amina scored 50 at pretest and fulfilled the Diagnostic and Statistical Manual of Mental Disorders, 4th edition (DSM-IV; American Psychiatric Association, 2000) criteria for PTSD with three criteria in each symptom clusters B–D. All scores are presented in Table 1. The University of California Los Angeles PTSD Reaction Index (UCLA-PTSD-RI; Steinberg, Brymer, Decker, & Pynoos, 2004) was completed by her (score 37) and Mrs. B (score 43; both critical range).

**Table 1:** Amina's symptom scores on all measures at pre, post, follow-up, and percentage improvement to baseline

				% Improvement	
				
Measure	Baseline	Post	6 months FU	Post	FU
CAPS-CA	50	6	5	**88%**	**90%**
UCLA-PTSD					
Self	37	20	6	45%	**84%**
Caregiver	43	18	36	**58%**	16%
CDI					
Self	21	16	7	24%	**67%**
SCARED					
Self	24	17	6	29%	**75%**
Caregiver	41	29	24	29%	41%

*Note*. CAPS-CA: Clinician Administered PTSD Scale for Children and Adolescents; UCLA-PTSD: University of California Los Angeles PTSD Reaction Index; CDI: Children's Depression Inventory; SCARED: Screen for Child Anxiety Related Disorders. Bold numbers indicate clinically reliable improvement (>50%; Ogles, 2013).

Furthermore, Amina completed the Children's Depression Inventory (CDI; Kovacs, 1985), a self-report on depressive symptoms (baseline score: 21, critical range). On the Screen for Child Anxiety Related Disorders (SCARED; Birmaher et al., 1999) Amina reported a score of 24 and Mrs. B of 41 (critical range). Even though the scores on self-report measures regarding depression and anxiety were elevated, Amina did not meet any diagnosis other than PTSD according to DSM-IV, as assessed with the Schedule for Affective Disorders and Schizophrenia for School-Age Children (K-SADS-PL; Kaufman et al., 1997).

All measures have previously been reported to have good psychometric properties in their German versions (UCLA-PTSD-RI: Ruf, Schauer, & Elbert, 2010; CAPS-CA: Steil & Füchsel, 2006; CDI: Stiensmeier-Pelster, Schürmann, & Duda, 2000; SCARED: Weitkamp, Romer, Rosenthal, Wiegand-Grefe, & Daniels, 2010). They were administered pre, post, and at the 6-month follow-up (FU) by a trained rater who was blind to treatment condition. Extended, written informed consent was given by Amina who is now 18 and, therefore, of age in Germany.

### TF-CBT

#### PRAC modules

In the first session, Amina received feedback on her baseline diagnostic results. The therapist (JU) offered psychoeducation on psychotherapy and PTSD in simple language and with the help of an illustrative graphic. Amina listed treatment goals together with the therapist: (1) be able to trust other people, (2) reduce intrusions, and (3) reduce hypervigilance and panic. In the caregiver session, Mrs. B also received feedback on the diagnostic results and psychoeducation. Mrs. B added emotion regulation as another goal as Amina's impulsiveness severely impaired her daily life.

In session 2, bodily tension as a result of PTSD symptoms and the rationale for relaxation were explained. As Amina reported intrusions when relaxing (e.g., at night in bed) and as she had panic-like attacks, focused breathing suited her needs best and was demonstrated and practiced together. As homework, Amina agreed to practice focused breathing daily. The same content was communicated to Mrs. B. Her homework involved informing and instructing the other caregivers and supporting Amina when she practiced.

When Amina arrived for session 3, she reported that she could look in the mirror again. She had tried it in combination with focused breathing and succeeded. The third session was dedicated to emotions. The therapist discovered that Amina did not know the names for different feelings in her mother tongue. She did, however, know how the basic emotions felt in her body and how other people would look if they felt a certain way. Therefore, the therapist decided to use pictures of emotional facial expressions (feeling face cards) to aid communication about emotions in this and each of the following sessions. In addition to being able to point towards a certain card, Amina practiced the names of the emotions. Coping strategies for unpleasant emotions were collected, tried in session and at home. Amina chose focused breathing as her strategy for anxiety and juggling three small balls for anger.

In session 4, the triangle of thoughts, feelings, and behavior was introduced to help Amina realize that thoughts influence feelings and behavior. She chose thoughts about school (“I will never succeed in learning German adequately”) and daily life in Germany (“I am ugly because my skin is not white”) to practice finding helpful cognitions. Mrs. B was shown the examples and instructed to support Amina in replacing negative cognitions with helpful alternatives.

#### T module

We started with a recapitulation of psychoeducation and the tools acquired so far (PRAC modules) and put these tools into a box to represent the preparation for creating the trauma narrative. Amina then drew a “life line” where she inserted all her good and bad experiences. She reported six traumatic events (according to the DSM-IV A criterion), four events that she did not report as traumatic, which however fulfilled the A1 criterion, and several chronic life stressors in her native country. She named school attendance in Germany as the only positive thing ever in her life. The therapist wrote the narrative while Amina was telling her story. She chose the deaths of her siblings and seeing their corpses as the worst moment. Additionally, Amina decided to write a chapter about her flight and her aunt's death. We, therefore, spent six sessions on the trauma narrative instead of the usual four. Focused breathing and juggling balls were used to manage her distress during the narration.

We identified dysfunctional cognitions concerning Amina's family (survivor's guilt, guilt for leaving them behind) and the issue of trust (nobody should be trusted). Amina succeeded in modifying those cognitions towards a more helpful and realistic perspective and added them to the narrative. She insisted on taking the narrative home and keeping it as part of her memory about her native country. She was so proud of it and convinced that one day she would write a real book about her life that we decided to let her take it home (some rules were agreed on, like keeping it in a safe place, only showing it to people you trust).

#### ICE modules

*In vivo* exposure was not necessary as Amina did not show severe avoidance of certain stimuli. She did, however, confront her fears by taking swimming lessons and even diving into the pool (her aunt had drowned). When asked about how she succeeded in doing this, she answered: “Breathe in, breathe out.”

In session 11, Amina shared her narrative with Mrs. B. She was very nervous about reading in German; however, she was also proud that she had written her story. Amina and Mrs. B asked each other questions that we had prepared in advance. They decided that they would create a memory book about Amina's native country and family together after the end of therapy.

In the last session, safety strategies were discussed and practiced (e.g., saying “no,” self-confident posture). When asked how the therapy had changed her, Amina answered: “I can now look in the mirror, I no longer hate myself, I am no longer afraid, and I can tell my story.”

#### Follow-up session

When Amina came in 6 months later, she brought her trauma narrative with her. She had added pictures of her mother and surviving sister and two paragraphs describing her arrival and the start of her life in Germany. She had also added some lessons learnt and some details about her life in her native country. She reported that she would be graduating a few weeks later and would go on to begin an apprenticeship.

### Outcome

Amina improved in a clinically reliable manner with regard to all measures. This was defined according to the Reliable Change Index (RCI; Jacobson & Truax, 1991) and the percentage improvement (Ogles, 2013). According to the CAPS-CA, she had no diagnosis of PTSD at posttreatment or follow-up (and no further diagnosis according to the K-SADS-PL). The percentage improvement was more than 50% for all measures for Amina's self-report at FU; her caregiver, however, reported less improvement. All outcome scores can be found in Table 1.

## Discussion

This case report demonstrates that evidence-based “western” treatment for PTSD can be successful with a young refugee who grew up in another culture and had limited language skills. All measures showed reliable improvement at the end of therapy and even further improvement at FU. Even though the caregiver gave the verbal feedback that the patient's symptomatology improved a lot and all therapy goals were attained, we did not find a clinically significant change on her measures. One possible explanation is that Amina turned 18 before FU which was accompanied by fears regarding deportation or exclusion from the group where she was living (due to reaching the age of majority). While Amina could distinguish these rational fears from trauma-related symptoms by filling in questionnaires with the rater, this was difficult for Mrs. B, who did so on her own.

The description of the therapy shows that no major modifications of the manual by Cohen et al. (2006) were necessary in this case. The minor additions to the manual – practicing names of emotions with face feeling cards over more sessions, drawing a life line, using two more sessions on the trauma narrative – do not reflect (cultural) modifications but rather a tailoring of the treatment to an individual patient. We would do this with any patient, regardless of culture, if necessary. To sum up, clinicians should not hesitate to treat young refugees, even with manualized approaches, as suggested by treatment guidelines (e.g., National Institute for Health and Care Excellence, 2005) especially if they are phase-based and culturally sensitive, as is TF-CBT (Cohen et al., 2006).

However, as this paper presents a single case, only limited conclusions can be drawn. It may be that Amina's cultural background was particularly compatible with the TF-CBT approach. More research is necessary to understand cultural differences in PTSD treatment, but this report shows that the blanket assumption that refugee minors will always need major adaptations to existing evidence-based interventions is not always warranted. In addition, our patient was very motivated and worked hard between the sessions and received a lot of social support in her residential group. We are aware that not every URM benefits from these circumstances.

## Supplementary Material

Case report: manualized trauma-focused cognitive behavioral therapy with an unaccompanied refugee minor girl
