# Supplementary material for: Case report: manualized trauma-focused cognitive behavioral therapy with an unaccompanied refugee minor girl
Source: Eur J Psychotraumatol. 2016 Jan 14;7:10.3402/ejpt.v7.29246. doi: 10.3402/ejpt.v7.29246 (PMC4716550; doi:10.3402/ejpt.v7.29246)
Supplement: Case report: manualized trauma-focused cognitive behavioral therapy with an unaccompanied refugee minor girl [file EJPT-7-29246-s001.pdf]

Fallbericht: Manualisierte traumafokussierte kognitive Verhaltenstherapie mit einem unbegleiteten minderjährigen Flüchtlingsmädchen

Johanna Unterhitzenberger, Rita Rosner

Hintergrund: Es besteht Unsicherheit, ob junge traumatisierte Flüchtlinge mit kulturell angepasster Therapie oder evidenzbasierten westlichen Ansätzen behandelt werden sollten. Bisher gibt es keine empirischen Studien für unbegleitete minderjährige Flüchtlinge in Industrienationen. Vorhandene Studien weisen aber darauf hin, dass eine traumafokussierte Behandlung für diese Patientengruppe vielversprechend ist.
Zielsetzung: Wir beschreiben die Behandlung eines unbegleiteten minderjährigen Flüchtlingsmädchens mit Posttraumatischer Belastungsstörung (PTBS), das manualisierte traumafokussierte kognitive Verhaltenstherapie erhielt (TF-KVT; Cohen, Mannarino & Deblinger, 2006).
Methode: Fallbericht. Ein 17-jähriges Mädchen aus Ostafrika, das ohne Erziehungsberechtigten nach Deutschland kam, wurde aufgrund ihrer PTBS behandelt. Diese resultierte aus mehreren traumatischen Erlebnissen in ihrem Heimatland sowie auf der Flucht. Sie lebte in einer Wohngruppe für Jugendliche. Es werden Daten vom Prä-, Post- und Follow-Up-Messzeitpunkt berichtet.
Ergebnisse: Das Mädchen nahm an 12 Sitzungen manualisierter TF-KVT teil; ihre Betreuerin aus der Wohngruppe erhielt in Anlehnung an das Behandlungsmanual weitere 12 Sitzungen. Es zeigte sich eine klinisch signifikante Symptomreduktion; nach der Therapie erfüllte das Mädchen die Diagnose PTBS nicht mehr. Der Therapieerfolg blieb über sechs Monate stabil.
Schlussfolgerung: Manualisierte TF-KVT ist anwendbar für junge Flüchtlinge ohne signifikante kulturelle Anpassungen. Der Ansatz kann aber als kultursensitiv angesehen werden.

Schlüsselbegriffe: Flüchtling, asylsuchend, getrennt, Jugendliche, PTBS, traumafokussiert, Behandlung. Psychotherapie, manualisiert

Citation: European Journal of Psychotraumatology 2015, **6**: 29246 - http://dx.doi.org/10.3402/ejpt.v6.29246
